# Identification of a phosphinothricin-resistant mutant of rice glutamine synthetase using DNA shuffling

**DOI:** 10.1038/srep15495

**Published:** 2015-10-23

**Authors:** Yong-Sheng Tian, Jing Xu, Wei Zhao, Xiao-Juan Xing, Xiao-Yan Fu, Ri-He Peng, Quan-Hong Yao

**Affiliations:** 1 Biotechnology Research Institute of Shanghai Academy of Agricultural Sciences, Shanghai, 201106, China; 2 Shanghai Ruifeng Agricultural Science and Technology Co., Ltd, Shanghai, 201106, China; 3 College of horticulture, Nanjing Agricultural University, Nanjing 210095, China; 4 College of horticulture, Shanxi Agricultural University, Taigu 030801, China

## Abstract

To date, only *bar/pat* gene derived from *Streptomyces* has been used to generate the commercial PPT-resistant crops currently available in the market. The limited source of *bar/pat* gene is probably what has caused the decrease in PPT-tolerance, which has become the main concern of those involved in field management programs. Although glutamine synthetase (GS) is the target enzyme of PPT, little study has been reported about engineering PPT-resisitant plants with *GS* gene. Then, the plant-optimized *GS* gene from *Oryza sativa* (*OsGS1S*) was chemically synthesized in the present study by PTDS to identify a *GS* gene for developing PPT-tolerant plants. However, *OsGS1S* cannot be directly used for developing PPT-tolerant plants because of its poor PPT-resistance. Thus, we performed DNA shuffling on *OsGS1S*, and one highly PPT-resistant mutant with mutations in four amino acids (A63E, V193A, T293A and R295K) was isolated after three rounds of DNA shuffling and screening. Among the four amino acids substitutions, only R295K was identified as essential in altering PPT resistance. The R295K mutation has also never been previously reported as an important residue for PPT resistance. Furthermore, the mutant gene has been transformed into *Saccharomyces cerevisiae* and *Arabidopsis* to confirm its potential in developing PPT-resistant crops.

L-Phosphinothricin [PPT; glufosinate; L-homoalanine-4-yl-(methyl)phosphinic acid] is the active ingredient of the nonselective herbicide BASTA (AgrEvo, Frankfurt am Main, Germany), which is a nonselective, postemergence herbicide used for weed control in orchards and vineyards^1^. PPT is a structural analog of glutamate that binds to glutamine synthetase (GS, E.C. 6.3.1.2), an enzyme responsible for the synthesis of glutamine from ammonia into glutamate^2^. Then, as glyphosate functioned by inhibiting its target enzyme: 5-enolpyruvylshikimate-3-phosphate synthase (EPSPS), PPT also act as an inhibitor of glutamine synthetase (GS) and eventually inactive GS irreversibly after phosphorylation at the active site of the enzyme^3^. As a consequence, the interaction between PPT and GS results in impairing amino acid metabolism and leading to plant death including some important economical crops^4^.

Recent advances in genetic engineering have rendered possible the transfer of PPT-tolerant genes to plants to produce PPT-tolerant crops, thereby ensuring that only weeds can be killed by PPT. Accordingly, two classes genes have been indentified and transformed into plants to engineer PPT-resistant plants. In the first, either the *bar* gene of *Streptomyces viridochromogenes* or the *pat* gene of *S. hygroscopicus*, which codes for the PPT acetyltransferase, has been transformed into plants to impart resistance to PPT^5^,^6^. Although presently there are more than 20 crop plant species transformed for resistance to PPT^7^, the transgene in most existing PPT-resistant transgenic crops is the *bar* or *pat* gene derived from a non-food species—*Streptomyces* bacteria. Thus, biosafety concerns are being raised in relation to the transformation of the heterologous PPT-resistant gene from pathogenic bacteria into crop plants. In another way, the recently discovery of PPT-resistant transgenic plant with an insensitive mutant *GS* gene resourced from plants has provided an alternative to the *bar/pat* gene of microorganisms for crop engineering^8^. Hence, obtaining novel and valid PPT-insensitive *GS* genes which is easily acceptable mentally by customer is critical for the development of PPT-resistant transgenic crops in future.

*In vitro*-directed evolution through DNA shuffling has been used frequently in directed evolution of therapeutic proteins, such as cytokines, enzymes, antibodies, and vaccines. As one of the most powerful tools for developing biologically active proteins, DNA shuffling can generate evolved recombinant proteins with improved enzyme kinetics^9^,^10^,^11^,^12^, altered substrate or product specificities^13^,^14^,^15^,^16^. Even more, the evolved enzyme with greate improvement of herbicide-resistance also can be created by DNA shuffling^17^.

To obtain novel and valid PPT-insensitive *GS* genes, the plant-optimized version of *GS* gene from *Oryza sativa* (*OsGS1S*) was chemically synthesized in the present study. Next, we performed DNA shuffling on *OsGS1S* gene under selective pressure caused by high PPT concentrations with the aim of obtaining mutants with improved PPT tolerence. Furthermore, the mutant gene has been transformed into *Saccharomyces cerevisiae* and *Arabidopsis* to confirm its potential in developing PPT-resistant crops.

## Result

### Synthesis of *OsGS1S* gene

**Figure 1:**
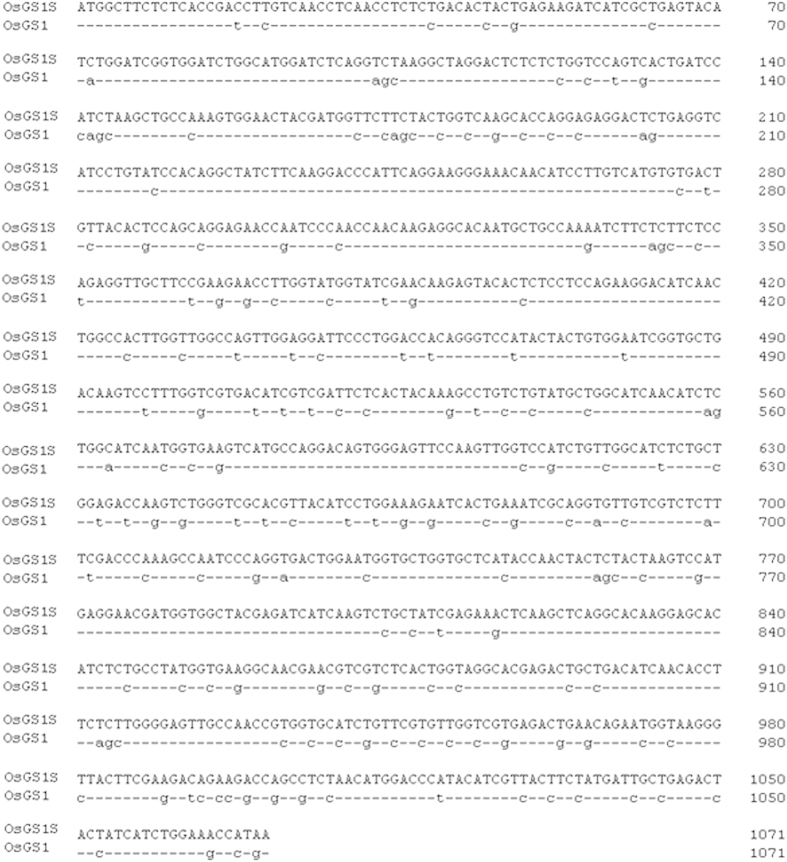
Comparation of the nucleotide sequence of *OsGS1* and *OsGS1S*.

Aiming to increase PPT-tolerant genes diversity and seek new PPT-tolerant genes for developing PPT-tolerant crops, the *OsGS1S* was artificially synthesized using PTDS stratege. BLAST search showed that the synthesized gene (*OsGS1S*) was 84.4% identical to the *OsGS1*. The A + T content of the *OsGS1* gene was 46.5%, whereas the A + T content of the (*OsGS1S*) gene was 49.9% because of the codon optimization (Fig. 1).

### Functional complementation analysis

**Figure 2:**
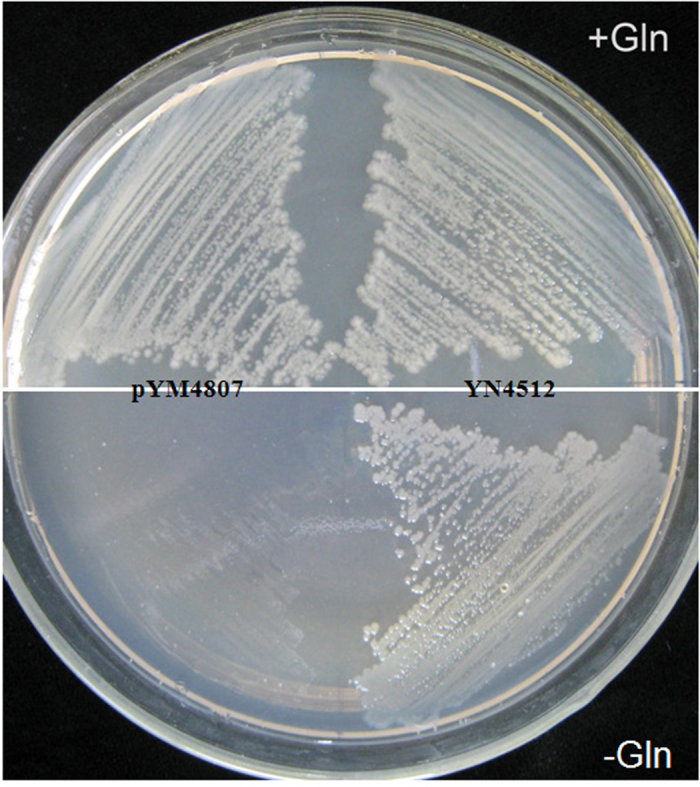
Functional complementation analysis. The EG82 strain was transformed with either pYM4087-OsGS1S (YN4512) or pYM4087. The pYM4087 plasmid was used as a negative control.

In order to assess whether the synthesized *OsGS1S* was functional in *E. coli*, heterologous complementation assays were performed. After 2 days incubation at 30 °C, the EG82 transformed with pYM4087-*OsGS1S* (pYN4512) grew well without glutamine added, whereas the EG82 transformed with the vector (pYM4087) alone could only grow in the presence of glutamine supplement, which means that the synthesized *OsGS1S* could complement the auxotrophic mutant by the functional production of glutamine (Fig. 2).

### DNA shuffling and sequencing

To obtain novel and mutant PPT-insensitive *GS* gene, the *OsGS1S* gene was shuffled using the DNA shuffling system. Over 500 000 variant colonies were screened using the pYPX251 vector in each iteration of DNA shuffling and screening. After three iteration of DNA shuffling, one mutant (OsGS1S_mutant_) was identified by its ability to restore growth in the mutant EG82 cell on M9 minimal medium containing 200 mM PPT. The mutant had mutations in 9 nucleic acid sites, resulting in alterations in 4 amino acids: A63E, V193A, T293A and R295K.

### Role of each altered amino acids in OsGS1S_mutant_

**Figure 3:**
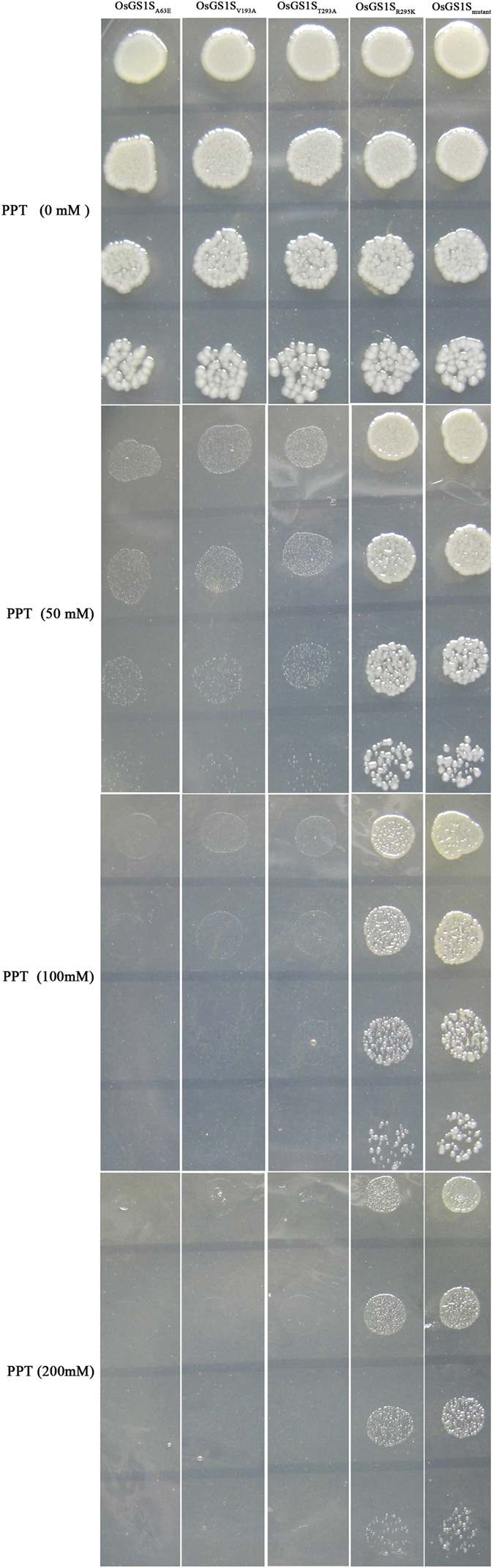
Drop test to determine the key amino acid mutations. Two μL of 1:5 serial dilutions were spotted on M9 medium with various PPT concentrations (0, 50, 100 and 200 mM). Data shown are representative of three independent experiments.

Site-directed mutagenesis and drop test were performed to determine the role of specific amino acids mutations in OsGS1S_mutant_. The results showed that the mutation R295K play a key role in improving PPT tolerance because cells carrying R295K mutation can proliferate on M9 minimal medium containing 200 mM PPT whereas no mutant with other’s mutations in A63E or V193A or T293A can proliferate at 50 mM PPT (Fig. 3). The findings suggested high PPT tolerance in these transformants assayed but only OsGS1S_R295K_, which confered higher tolerance to PPT.

### Kinetic properties

To further verify the key role of R295K mutations in OsGS1S_mutant_, the OsGS1S, OsGS1S_mutant_ and OsGS1S_R295K_ were overexpressed and purified in *E. coli* (S Fig. 1). The obtained kinetic constants are shown in Table 1. The *K*_*i*_ for OsGS1S_R295K_ was 192.5 μM which is similar to OsGS1S_mutant_ whereas the *K*_*i*_ of OsGS1S was only 2.2 μM. *K*_*m*_, a measure of the affinity for substrate, was 1.22, 8.04 and 7.99 mM for OsGS1S, OsGS1S_R295K_ and OsGS1S_mutant_, respectively. This showed that the substrate binding affinities was only slightly decreased for the OsGS1S_R295K_ and OsGS1S_mutant_. So the results further imply that only single-site mutation, OsGS1S_R295K_, is sufficient to enable PPT resistance and maintain relatively high affinity for the substrate glutamate.

### *In vitro* PPT sensitivity assays in yeast

**Figure 4:**
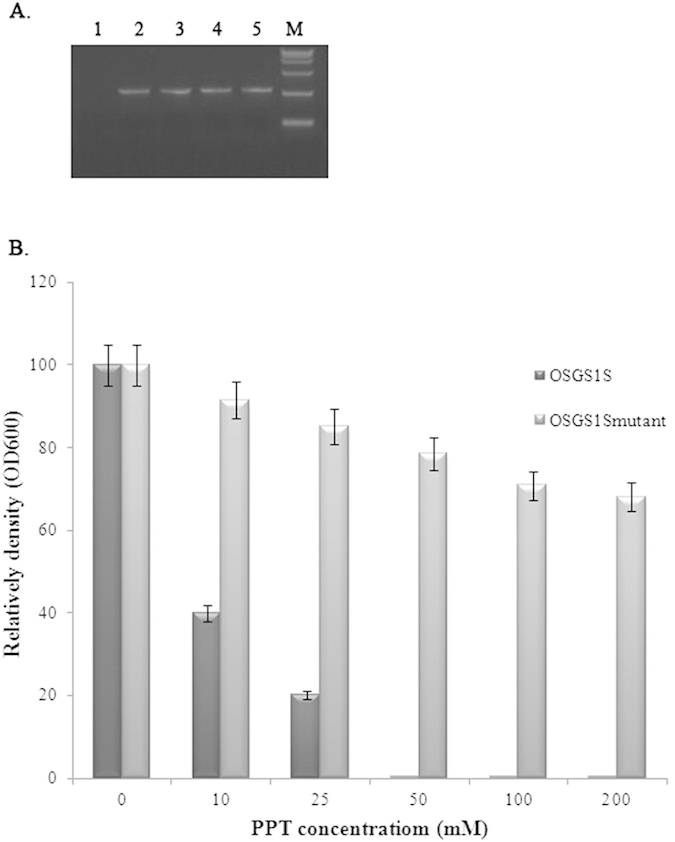
(**A**) Molecular analysis of transformation using DNA as template; 1: non-transformed; 2 and 3: EGY48 transformed with *OsGS1S*_mutant_; 4 and 5: EGY48 transformed with *OsGS1S*; M: Takara marker 15000 bp. (**B**) *In vitro* PPT sensitivity assays of yeast transformation at various PPT concentrations (0, 10, 25, 50, 100 and 200 mM).

*In vitro* PPT sensitivity assays of the EGY48 harboring plasmid pYF1274-*OsGS1S*_muatnt_ or pYF1274-*OsGS1S* were used to determine if OsGS1S_muatnt_ conferred PPT resistance in yeast. Firstly, to verify whether the *OsGS1S* or *OsGS1S*_mutant_ has been integrated into yeast genome, the transformed clone and the non-transformed clone (CK) were analyzed for nuclear integration by PCR. The target fragments were amplified from the genomic DNA of the transformed clone. No target DNA bands was revealed from CK clones (Fig. 4A). Thus, the PCR analysis confirmed that *OsGS1S* and *OsGS1S*_mutant_ gene were present in their transformations.

Then, the positively transformed clones were selected for PPT sensitivity assays. As shown in Fig. 4B, after 36 h incubation, the growth of cells harboring pYF1274-*OsGS1S* was strongly inhibited ( >98%) by 50 mM PPT whereas the OD_600_ of cultures harboring pYF1274-*OsGS1S*_muatnt_ was ~78% compared with controls without PPT. At 100 mM PPT concentrations, cultures harboring pYF1274-*OsGS1S*_muatnt_ still attained OD values that were ~70% of control values but growth was slightly inhibited ( >30%) at 200 mM PPT. It is obvious that OsGS1S_mutant_ is more tolerant of PPT than OsGS1S.

### Transgenic plant selection

Transgenic *Arabidopsis* was used to evaluate the potential application of OsGS1S_mutant_ in developing PPT-resistant crops. Two OsGS1S (W1 and W2) and two OsGS1S_mutant_ (M1 and M2) transgenic lines were analyzed for gene expression using RT-PCR analysis as previously described^18^. Agarose gel electrophoresis showed that the DNA intensity of *OsGS1S* and *OsGS1S*_mutant_ in the four different transgenic lines was the same (S Fig. 2), confirming that the level of transcription of *OsGS1S* and *OsGS1S*_mutant_ in the two transformants was equal. The OsGS1S transgenic line W1 and the OsGS1S_mutant_ transgenic line M2 were chosen for further experiments.

### Assay for the PPT resistance of transgenic *Arabidopsis*

**Figure 5:**
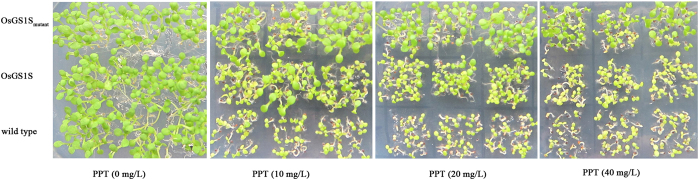
Comparative germination image of OsGS1S_mutant_-transgenic plants, OsGS1S-transgenic plants and wild type (WT) plants at various PPT concentrations (0, 10, 20 and 40 mg/L) in Petri dishes.

**Figure 6:**
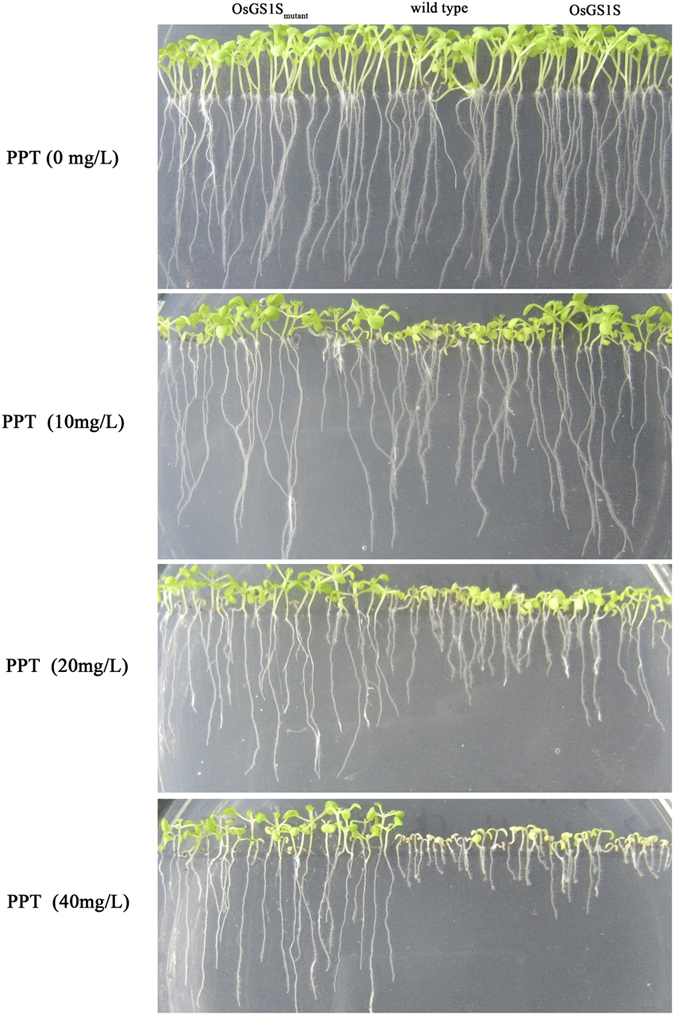
Comparative root growth image of OsGS1S_mutant_-transgenic plants, OsGS1S-transgenic plants and wild type (WT) plants at various PPT concentrations (0, 10, 20 and 40 mg/L) in Petri dishes.

A previous study has reported that seeds and roots are usually poorly developed under PPT stress^19^. Hence, the assay of root growth and germination was performed and morphological difference among different plants was shown in Figs 5 and 6. After 2 weeks, the root growth of OsGS1S_mutant_-transgenic plants was able to grow at 40 mg/L PPT, whereas OsGS1S-transgenic plants and wild type (WT) plants were strongly inhibited at 20 mg/L. It is obvious that OsGS1S_mutant_-transgenic plants were more tolerant to PPT than OsGS1S-transgenic plants and WT plants, which was further confirmed by the germination assay of these plants and transgenic rice. (S Fig. 3). The OsGS1S_mutant_-transgenic plants grew well at 40 mg/L PPT, whereas the OsGS1S-transgenic plants and WT did not.

**Figure 7:**
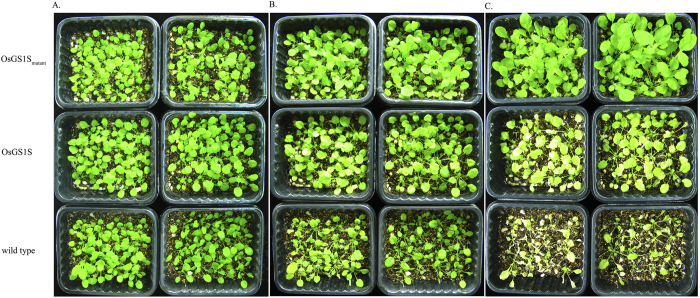
Photographs of the transgenic plants sprayed with BASTA solution (150 mg/L active ingredient). The same results were obtained in three independent experiments and are represented by the effects shown here. (**A**) before sprayed with BASTA solution. (**B**) seven days after firstly sprayed with BASTA solution. (**C**) seven days after secondly sprayed with BASTA solution.

A previous study also showed that lethal concentrations of PPT caused yellowish-brow symptoms on leaves^19^. This indicates that the phenomenon of yellowish-brown leaves is a distinguishing characteristic of plants exposed to lethal PPT concentrations. After 3 weeks, the seedlings in pots were twice treated with PPT-based BASTA solution (150 mg/L active ingredient) at 7-day intervals. As showed in Fig. 7, seven days after the first application using BASTA solution, the leaves of the WT plants turned yellowish-brown and minor damages appeared in the OsGS1S-transgenic plants. After spraying for the second time, most leaves of the WT turned severely yellowish-brown even to dehydration and death, the OsGS1S-transgenic plants also turned yellowish-brown, but the OsGS1S_mutant_-transgenic plants grew well with normal morphology. These results also indicate that OsGS1S_mutant_-transgenic adult plants are more resistant to PPT exposure than OsGS1S-transgenic plants and WT plants.

## Discussion

The main objective of our study was to identify a mutant *GS* with properties appropriate for the development of transgenic PPT-tolerant plants. However, wild-type *OsGS1S* cannot be directly used for the development of transgenic PPT-tolerant plants because of its extreme PPT sensitivity. Hence, we performed DNA shuffling on *OsGS1S*, and one highly PPT-resistant mutant with four amino acids variations was isolated after three rounds of DNA shuffling and screening. Among the four amino acids substitutions on this mutant, only one residue change was identified by site-directed mutagenesis as essential in altering PPT resistance.

The crystal structure of plant GS has been determined at 2.63-, 3.50- and 3.80-A resolutions, respectively^20^. And the enzyme has two face-to-face pentameric rings with a total of 10 active sites, each formed with the N-terminal domain of one subunit and the C-terminal domain of the neighboring within each ring^20^. Based on what is known about the crystal structure of plant GS, some mutation of amino acids in active sites result in significant change in PPT tolerance have been studied and identified. For example, alteration at E254D in the GS enzyme of *Zea mays* has been shown to confer resistance to glufosinate (PPT)^8^. Meanwhile, another specific mutation of amino acids which not positioned in active sites but at a position distant from the active site can also alter PPT resistance. For example, mutations such as D56E may result in important changes in the *K*_*m*_ for glutamate in α-GS from *Phaseolus vulgaris*^21^.

A new mutation of OsGS1S, R295K, that also affects PPT resistance has been identified in the present study. To better understand the functions of this amino acid substitution, the mutation in OsGS1S_mutant_ was distributed throughout the structure models of OsGS1S based on the crystal structures of GS of *Zea mays*. The three-dimensional structures suggested that R295K is situated at N-terminal domain of one subunit. More specifically, it is located between two crucial sites—Arg291 and Arg311, the side chains of which are bond with phosphinothricin phosphate and phosphorylated MetSox through hydrogen bond interactions inaddition to three Mn^2+^ ions^20^. Therefore, although R295K is not directely involved in the substrate binding, considering that it is within the substrate binding region, any amino acid substitution in this region that indirectly affects the hydrogen bonding networks of this region may weaken the phosphinothricin phosphate binding to the enzyme complex, faslitate binding of glutamate, and potentially result in difference in enzyme behavior. Thus, upon replacement of Arg 295 by Lys in OsGS1S may relatively altered the hydrogen bonding networks and thus easily bind with glutamate but not PPT. Therefore, the affinity of the enzyme for PPT decreased and the PPT-torlence improved which was further confirmed by the *K*_*i*_value and the assay of PPT resistance in yeast.

The development of plant resistant to PPT has been a goal in the engineering of many plant species. In 1987, the firstly transformation of PPT-resistant plants has been accomplished by DeBlock *et al*.^6^. Since then, many crop plant species have been engineered for PPT resistance, including rice, soybean, maize, wheat, tomato, carrot, cotton, sorghum and potato *et al*. Unfortunately, the transgene in most existing PPT-resistant crops is the *bar* or *pat* gene derived from *Streptomyces*. In addition to biosafety concerns, the limited source of *bar/pat* gene is probably what has caused the decrease in herbicide tolerance, which has become the main concern of those involved in field management programs. Increasing PPT-resistant gene diversity and generating multi-herbicide-resistant crops is an effective approach to this problem^22^. Although GS is the target enzyme of PPT, little study has been reported about engineering PPT-resisitant transgenic plant with *GS* gene. As with the same issue for engineering glyphosate-resisitant transgenic plant, the main obstacle has to be overcome for development of PPT-tolerant palnts with *GS* gene is identifying effective PPT-insensitive *GS* genes. In the present study, we represented a successful generation of novel OsGS1S_mutant_ transgenic *Arabidopsis*. Our results showed that OsGS1S_mutant_ can confer transgenic *Arabidopsis* plants with relatively high tolerance to PPT. Although, the resistance level of the OsGS1S_mutant_-transgenic *Arabidopsis* is not as high as reported level^7^, our study demostrated that the insensitive mutant *GS* gene could be a good application candidate in developing crops with PPT tolerance in future.

## Materials and Methods

### Strain and vectors

The *E. coli* Gln-auxotrophic strain JW3841-1 (glnA732::Kan) was obtained from the *E. coli* Genetic Resource Center (CGSC#10775, Yale University, USA). The pRARE plasmid (Novagen, USA), which over-expresses tRNAs for Arg, Leu, and Pro codons rarely used by *E. coli*, was introduced into JW3841-1 (namely EG82) to enhance the expression of GS. The expression vector pYM4087^23^ which contains a strong constitutive tac1 promoter and a six-histidine tag, was constructed in our laboratory. The yeast expression vector pYF1274 (S Fig. 4) which contains a strong PGK promoter and ADC1 terminator, was constructed in our laboratory.

### Design and chemical synthesis of *OsGS1S* gene

The sequence of *OsGS1S* was artificially synthesized using a PCR-based two-step DNA synthesis (PTDS) strategy^24^,^25^ according to the sequence of cytosolic glutamine synthethase from *Oryza sativa* (*OsGS1*,GenBank:AB037595). All the codons in *OsGS1S* were optimized and preferentially designed for plants^26^. PCR was carried out in a total volume of 50 μL containing 200 nM of each outer primer and 20 nM of each inner primer. The reaction conditions were 30 cycles of 94 °C for 30 s, 54 °C for 30 s, and 72 °C for 90 s. The amplified fragment was digested with BamHI/SacI, cloned into simple pMD 18-T and sequenced. Errors in the synthetic gene were corrected by the overlap extension PCR method^24^.

### Functional complementation analysis

For complementation analysis, the *OsGS1S* was introduced into EG82 strain. Subsequently, the transformants were streaked on M9 minimal medium in the presence or absence of 1 mM L-glutamine and grown at 30 °C for 2 days. EG82 strain harboring the empty vector plasmid was used as a control.

### DNA shuffling

DNA shuffling to generate improved PPT-tolerance mutants was performed as described by Stemmer *et al*.^27^ and Xiong *et al*.^28^. The *OsGS1S* was fragmented with DNase I, and fragments from 50 to 100 bp were collected in a dialysis bag as they eluted from 10% (*w*/*v*) polyacrylamide electrophoresis gels. The fragments were subjected to PCR reassembly without primers. Then the “primeless” PCR products were subjected to PCR with the specific primers P1Z (5′-gagagaggatccatggcttctctcaccga-3′) and P1F (5′-gagagagctctcatggtttccagatga-3′) to amplify the *OsGS1S* to full length. After the primerless PCR and primer PCR, a group of full-length *OsGS1S* mutants were obtained and digested with BamHI/SacI. The isolated fragments were ligated into the prokaryotic expression vector pYPX251^28^. The mutant DNA library was translated into EG82 by electroporation, and was plated on M9 agar plates supplemented with PPT at various concentrations. The *OsGS1S* mutant was screened and identified by its ability to restore the growth of EG82 cells on M9 minimal medium containing PPT at high concentrations. The *OsGS1S* mutants were pooled and used as templates to generate further mutants by DNA shuffling. The selected *OsGS1S* mutants were sequenced and subsequently analyzed.

### Site-directed mutagenesis and identity of key amino acids mutation

To determine the role of specific amino acid mutations in the mutant, site-directed mutagenesis was performed. Four had individual amino acids mutations (OsGS1S_A63E_, OsGS1S_V193A_, OsGS1S_T293A_ and OsGS1S_R295K_) were created using an overlap extension PCR strategy^25^. The used primers are: A63E, (5′-ggtcaagaaccaggagaggac-3′) and (5′-tcctggttcttgaccagtaga-3′); V193A, (5′-ggtgaagccatgccaggacag-3′) and (5′-tggcatggcttcaccattgat-3′); T293A (5′-cgtctcgctggtaagcacgag-3′) and (5′-cttaccagcgagacgacgttc-3′); R295K, (5′-gctggtaagcacgagactgct-3′) and (5′-ctcgtgcttaccagcgagacg-3′).

To identity the key amino acids mutation, EG82 transformants (transformed with OsGS1S_A63E_ or OsGS1S_V193A_ or OsGS1S_T293A_ or OsGS1S_R295K_) were grown in LB medium. After induction in LB medium for 24 h, cells were diluted to 5 × 10^3^ cells/μL. About 2 μL of 1:5 serial dilutions were spotted on fresh M9 agar plates supplemented with PPT at various concentrations (0, 50, 100 and 200 mM) and then incubated at 30 °C for 2 days.

### Protein overexpression and purification

The OsGS1S, OsGS1S_R295K_ and OsGS1S_mutant_ digested with BamHI/SacI were cloned into the expression vector pYM4087. They were then expressed and introduced into the competent expression host, *E. coli* BL21 (DE3). Bacteria were then plated on LB medium. The culture was incubated at 30 °C. When the OD of cultures was 0.7 at 600 nm, cells were collected by centrifugation and resuspended in 4 ml s of lysis buffer (50 mM Tris-HCl, pH 7.5, 300 mM NaCl, 0.5 mM dithiothreitol [DTT], and 10 mM imidazole). The His-tagged protein in the soluble fraction was affinity-purified using a nickel-nitrilotriacetic acid agarose column (1.5 ml bed volume) (sigma). Washing was carried out with a buffer containing 50 mM Tris-HCl, pH 7.5, 300 mM NaCl, and 50 mM imidazole. Protein was eluted with the same buffer containing 500 mM imidazole. The GS fractions were then dialyzed against buffer (50 mM Tris-HCl, pH 7.5, 5 mM MgCl_2_, and 10% glycerol). The purified protein was analyzed by 12% SDS-PAGE and protein concentration was quantified using Bradford assay kit and bovine serum albumin (BSA) as standard.

### Enzyme assay

In general, GS activity was measured using a semibiosynthetic assay modified from that of Listrom *et al*.^29^. The semibiosynthetic assay mixture (total volume 80 μl) contained: 5 mM ATP, 20 mM L-glutamate, 30 mM hydroxylamine, 50 mM MgCl_2_ and 20 μl of the enzyme solutionin 100 mM Tris-HCl (pH 7.5). The mixture was equilibrated at 37 °C for 5 min, and the reaction was initiated by adding 20 μl of the enzyme solution. The reaction was stopped after 30 min by the addition of 100 μl of ferric chloride reagent (55 g/L FeCl_3_·6H_2_O, 20 g/L trichloroacetic aci, and 2.1% concentrated HCl) and the release of γ-glutamyl hydroxamate was quantified by measuring the absorbance at 540 nM. One unit of enzyme activity was defined as the amount of enzyme that catalyzed the formation of 1 μmol of γ-glutamyl hydroxamate per minute^30^.

The *K*_m_ values for glutamate and the *K*_i_ values for PPT were measured as described by Tian *et al*.^31^.

### Yeast transformation, molecular analysis of transformation and *In vitro* PPT sensitivity assay

The *OsGS1S* and *OsGS1S*_muatnt_ were digested by BamHI/SacI and subcloned into BamHI/SacI-digested pYF1274 expression vector. Accordingly, the recombinant plasmids were transformed into *S. cerevisiae* strain EGY48 (MATα, his3 trp1 ura3-52leu::pLeu2-LexAop6) by using the lithium acetate method^32^. The transformants were cultured on SD plates at 30 °C for 3 d.

To verify the integration of target genes into *S. cerevisiae* genome, the genomic DNA of transformed clones or non-transformed clones (CK) was extracted according to the published method^32^. Then, using the yeast genomic DNA as a template, PCR was carried out. The PCR-amplified DNA fragments were separated by electrophoresis on 1% agarose gel and visualized under UV irradiation.

The positively transformed clones were selected and gorwn at 30 °C in liquid M9 minimal medium for *In vitro* PPT sensitivity assays. When the OD_600_ reached 0.2, inocula of the two transformants containing equal amounts of the target protein were transferred to fresh 5 ml aliquots of liquid M9 minimal medium supplemented with PPT at various concentrations ranging from 0 to 200 mM. Cultures were incubated as above and OD_600_ values determined after 36 h.

### Plant expression vector construction and plant transformation

Construction of the plant expression vector and plant transformation was performed as described by Xu *et al*.^18^. The *OsGS1S* and *OsGS1S*_muatnt_ were digested with BamHI/SacI, and the fragment was inserted between the double CaMV 35S (D35S) promoter and nopaline synthase terminator (NOS-Ter) of plant expression vector pYK4102. The recombinant plasmid was introduced into *Agrobacterium tumefaciens* GV3101. The constructions were transformed into *Arabidopsis* (Col.) by floral dip method as described previously^33^. Transgenic plants were selected by hygromycin resistance and confirmed by PCR using the primers mentioned above.

### Transgenic plant selection and transcript analysis

The transgenic nature of the plants was further confirmed by PCR analysis of genomic DNA using the specific primers P1Z and P1F. Reverse transcription (RT)-PCR was used to determine the level of *OsGS1S* or *OsGS1S*_mutant_ transcription. In order to improve the reliability of RT-PCR, the *Arabidopsis* actin gene (GenBank: U41998) served as an internal standard to normalize the amount of cDNA was amplified with primers TactZ (5′-gcaccctgttcttcttaccgag-3′) and TactF (5′-agtaaggtcacgtccagcaagg-3′). Specific DNA fragments (~1100 bp) of the *OsGS1S* or *OsGS1S*_mutant_ gene were then amplified from the transgenic plants using the same amount of cDNA. PCR products were separated on 2% (w/v) agarose gels and quantified using a Model Gel Doc 1000 (Bio-Rad, USA). The expression patterns of the *OsGS1S* or *OsGS1S*_mutant_ genes were evaluated with a Shine Tech Gel Analyzer (Shanghai Shine Science of Technology Co., Ltd., China).

### Assay of PPT resistance in transgenic *Arabidopsis*

For the assay of germination, the T_3_-sterilized *A. thaliana* seeds were grown directly on MS medium containing various PPT concentrations (0, 10, 20 and 40 mg/L) in Petri dishes under a controlled-environment chamber (22 °C, 16:8 h day:night cycle). To observe the germination process, photos were taken after 2 weeks of growth.

For the assay of root growth, the T_3_-sterilized *A. thaliana* seeds were vertically grown on MS medium containing various PPT concentrations (0, 10, 20 and 40 mg/L) in Petri dishes in a controlled environment chamber at 22 °C kept on a 16/8 h day/night cycle. After grown vertically for 2 weeks, the photos were taken.

For the PPT spray treatment, plants were grown in pots (16 seedlings per pot) filled with 9:3:1 vermiculite:peat moss:perlite in a controlled-environment chamber (22 °C, 16:8 h day:night cycle,). After 3 weeks, the seedlings in the pots were twice treated with PPT-based BASTA solution (150 mg/L active ingredient) at 7-day intervals. Then, photos were taken at 7-day intervals.

## Additional Information

**How to cite this article**: Tian, Y.-S. *et al*. Identification of a phosphinothricin-resistant mutant of rice glutamine synthetase using DNA shuffling. *Sci. Rep*. **5**, 15495; doi: 10.1038/srep15495 (2015).

## Supplementary Material

Supplementary Information

## Figures and Tables

**Table 1:** The values of *K*_m_ and *K*_i_ for OsGS1S, OsGS1S_mutant_ and OsGS1S_R295K_.

	*K*_m_ (L-glutamate, mM)	*K*_i_(PPT, μM)
OsGS1S	1.22 ± 0.011	2.2 ± 0.010
OsGS1S_mutant_	7.99 ± 0.021	193.4 ± 0.014
OsGS1S_R295K_	8.04 ± 0.015	192.5 ± 0.022
