# Supplementary material for: Identification of a phosphinothricin-resistant mutant of rice glutamine synthetase using DNA shuffling
Source: Sci Rep. 2015 Oct 23;5:15495. doi: 10.1038/srep15495 (PMC4616025; doi:10.1038/srep15495)

**Identification of a phosphinothricin-resistant mutant of rice glutamine synthetase using DNA shuffling**

Yong-Sheng Tian^1,2,3^, Jing Xu^1^, Wei Zhao^1^,Xiao-Juan Xing^4^, Xiao-Yan Fu^1^, Ri-He Peng^1^*****, Quan-Hong Yao^1^*****

# *1: Biotechnology Research Institute of Shanghai Academy of Agricultural Sciences, Shanghai, 201106, China*

*2:Shanghai Ruifeng Agricultural Science and Technology Co., Ltd, Shanghai, 201106, China*

*3: College of horticulture, Nanjing Agricultural University, Nanjing 210095, China*

*4: College of horticulture, Shanxi Agricultural University, Taigu 030801, China*

Running head: A mutant *GS* from *Oryza sativa* by DNA shuffling

***Corresponding author:**

*Dr. Quanhong Yao*

*Director and Professor*

*Shanghai Key Laboratory of Agricultural Genetics and Breeding*

*Biotechnology Research Institute*

*Shanghai Academy of Agricultural Sciences*

*2901 Beidi Road, Shanghai, People’s Republic of China*

*Tel.: +86-21-62203180; Fax: +86-21-62203180*

*Email address: tys810508@126.com*

Yong-Sheng Tian and Jing Xu are contributed equally to the article

**Fig.1.** SDS-PAGE analysis of recombinant OsGS1S, OsGS1S_mutant_ and OsGS1S_R295K_. The purified recombinant enzymes wrer subjected to SDS-PAGE on 0.1% SDS-12% PAGE and stained with Coomassie Brilliant Blue R-250. *M*, Protein Molecular Weight Marker (Low, Takara); *lane 1*, *OsGS1S*; *lane 2* *OsGS1S*_mutant_; *lane 4,* OsGS1S_R295K_.


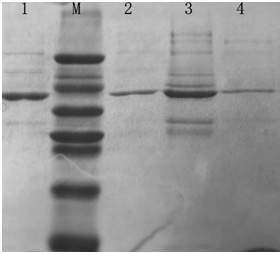


**Fig.2.** Expression of *OsGS1S*_mutant_ and *OsGS1S* cDNAs in different transformants. Each lane contained 4 μL of RT-PCR products obtained using total RNA extracted from three-week-old plants grown under normal conditions. CK, wild type；W1 and W2, transgenic *Arabidopsis* lines with *OsGS1S* gene; M1 and M2, transgenic *Arabidopsis* lines with *OsGS1S*_mutant_ gene. Data shown are representative of three independent experiments


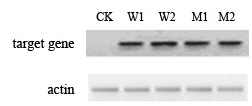


**Fig.3.** The T_1_ generation sterilized rice seeds were used for seed germination assay. A lot of 12 individuals of each transgenic line were plated directly on each region of the Petri dishes containing half-strength **MS** with various PPT concentrations (0, 5mg/L, and 10 mg/L) under a controlled-environment chamber (25°C, 10:14 h day:night cycle). Then, germination percentage was scored after 1 weeks of growth and photos were taken. WTr: transgenic rice lines with wild type OsGS1S gene; Mur: transgenic rice lines with OsGS1_mutant_ gene.


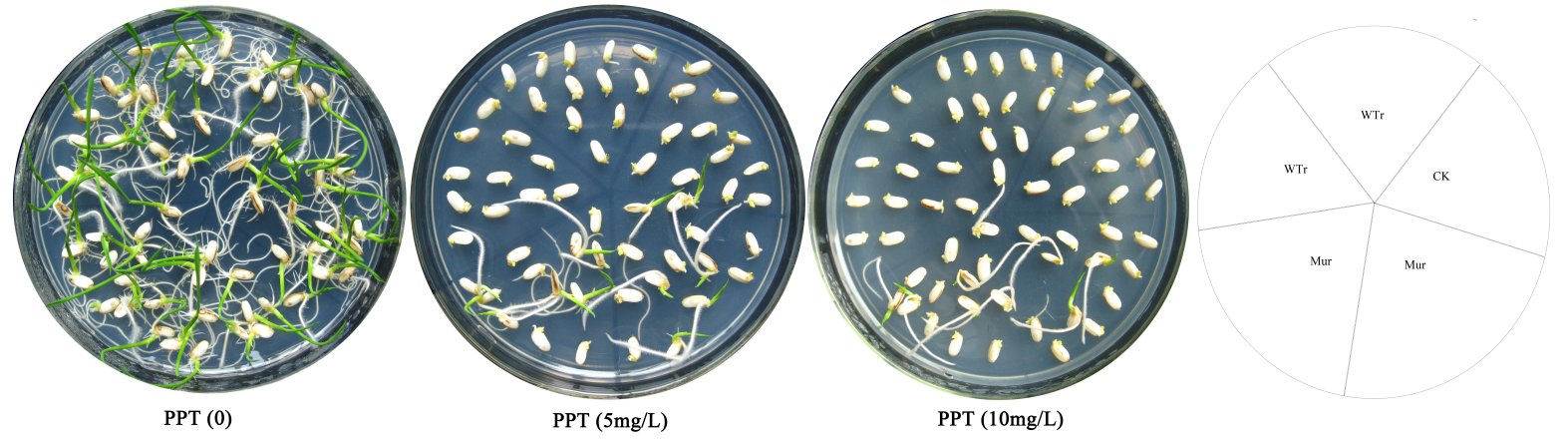


**Fig.4.** Schematic diagram of the pYF1274 vector


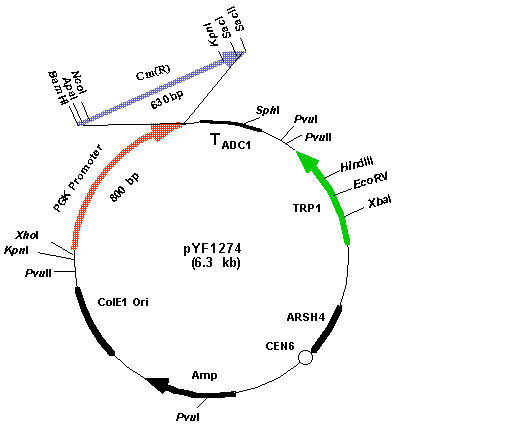

Supplement: Supplementary Information [file srep15495-s1.doc]
